# The supplementation of L-carnitine in septic shock patients: Systematic review and meta-analysis

**DOI:** 10.1016/j.clinsp.2022.100124

**Published:** 2022-10-31

**Authors:** Gabriel Voltani Guedes, Marcos Ferreira Minicucci, Suzana Erico Tanni

**Affiliations:** a Faculdade de Medicina de Botucatu, Universidade Estadual Paulista (UNESP), Botucatu, SP, Brazil; b Internal Medicine Department, Faculdade de Medicina de Botucatu, Universidade Estadual Paulista (UNESP), Botucatu, SP, Brazil

**Keywords:** L-carnitine, Septic shock, Mortality, Mitochondrial dysfunction

## Abstract

**Background:**

Sepsis and septic shock are severe and difficult-to-treat conditions with high lethality. There is interest in identifying new adjunct therapies that are effective in reducing mortality. In this context, L-carnitine has been investigated in trials as a potentially beneficial drug. Therefore, the aim of this systematic review was to assess the clinical evidence to support the use of L-carnitine in septic shock patients to reduce the risk of mortality. The objective of this review was to evaluate the effect of L-carnitine compared to placebo or Usual Care (UC) on the mortality rate in hospitalized adult septic shock patients.

**Methods:**

The authors exclusively included randomized clinical trials that compared the use of L-carnitine versus placebo in adult (> 18 years old) septic shock patients. The outcome was a mortality rate of 28 days. This systematic review and meta-analysis were performed following the PRISMA guidelines and registered in PROSPERO with the ID CRD42020180499.

**Results:**

Following the initial search, 4007 citations were identified, with 2701 remaining after duplicate removal. Eight citations were selected for body text reading, and two were selected for inclusion. The studies enrolled 275 patients, with 186 in the carnitine arm and 89 in the placebo arm. The effect of L-carnitine uses in septic shock patients showed a difference risk of -0.03 (95% Confidence Interval: -0.15–0.10, I^2^ = 77%, p = 0.69) compared to placebo/in mortality rate with low quality of evidence.

**Conclusions:**

There is low-quality evidence that the use of L-carnitine has no significant effect on reducing 28-day mortality in septic shock patients.

## Highlights

• The objective of this review was to evaluate the effect of L-carnitine compared to placebo or Usual Care (UC) on the mortality rate in hospitalized adult septic shock patients. •Two Randomized Controlled Trials were selected for inclusion in this review •There is low-quality evidence that L-carnitine has no significant effect on 28-day mortality of septic shock patients

## Background

Sepsis can be defined as organ dysfunction resulting from the dysregulation of the host's response to infection. Septic shock is a subset of sepsis with circulatory, cellular, and metabolic abnormalities that substantially increase mortality. Septic shock has a high cost of treatment, with an increasing incidence and high mortality.^1^ Brazilian data show that in the period from 2006 to 2015, the incidence grew by 50.5%, representing a rate of 47.4/100,000 people per year, with a mortality rate ranging from 43% to 60%.^2^

The pathophysiology of sepsis is complex and still not fully understood. However, metabolic abnormalities are well-documented in patients with sepsis and septic shock. The main alterations include hyperglycemia, hyperlactatemia, ketosis, and increased levels of free fatty acids, and acylcarnitines.^3^, ^4^, ^5^, ^6^, ^7^, ^8^ Inhibition of the pyruvate dehydrogenase complex leads to anaerobic metabolism and increased lactate production^9^^,^^10^ and is considered a strong predictor of mortality in these patients. In addition, patients with sepsis have increased urinary secretion of carnitine. Carnitine deficiency leads to carnitine palmitoyl transferase 1 dysfunction and alterations in the beta-oxidation of fatty acids.^11^^,^^12^

Currently, international guidelines for septic shock advocate early diagnosis and treatment with fluid resuscitation, early introduction of vasopressors and antibiotics, glycemic control, and corticosteroid usage.^13^ Despite these strategies, the mortality rate remains extremely high. Therefore, research on potential treatments that can improve prognosis is mandatory. One of these treatments is L-carnitine. L-carnitine enhances fatty acid entry into the mitochondria, mitigating their toxic effects in the cytosol, and decreasing the inhibitory effect of acetyl-coenzyme A on the pyruvate dehydrogenase complex caused by intramitochondrial acetate.^14^

Studies that evaluate L-carnitine supplementation in critically ill patients suggest that it reduces inflammatory biomarkers compared to placebo.^15^^,^^16^ In addition, serum levels of carnitine and its esters in patients with sepsis and septic shock are strong predictors of patient mortality. However, the role of L-carnitine supplementation in patients with septic shock is still not well understood. Therefore, the aim of this study was to perform a systematic review and meta-analysis to evaluate the effect of L-carnitine compared with placebo on mortality in patients with septic shock.

## Methods

This systematic review was conducted following the Preferred Reporting Items for Systematic Reviews and Meta-Analyses (PRISMA) recommendations.^17^

### Inclusion criteria

The protocol of this study was based on the Patients of interest, Intervention to be studied, Comparison of intervention, and Outcome of interest (PICO) methodology. Regarding the use of L-carnitine, the PICO framework was as follows: patients, adult septic shock patients (> 18 years old); intervention, use of L-carnitine by any route of administration; comparison, Usual Care (UC) or placebo; and outcome, mortality for any cause in 28 days. Intermediate outcomes, such as hospitalization days, were excluded. The eligibility criteria for the inclusion of studies were Randomized Controlled Trials (RCTs) of at least 28 days. The authors imposed no restrictions on the date of publication, language, or full-text availability. For inclusion, studies must have reported the definition of septic shock as a life-threatening organ dysfunction caused by a dysregulated host response to infection.^18^ This review has been registered on PROSPERO under registration number CRD42020180499.

### Search strategy

The search strategy was based on published and unpublished studies. An initial limited search of MEDLINE was undertaken to identify articles on the topic. The text words contained in the titles and abstracts of relevant articles and the index terms used to describe the articles were used to develop a full search strategy for MEDLINE via Medline, EMBASE, LILACS, Scopus, Cochrane database, and Universidade de São Paulo thesis database. The search strategy, including all identified keywords and index terms, was adapted for each database. The reference lists of all the included sources of evidence were screened for additional studies. Specific search strategies were used for each database. The search strategy included studies published until November 11, 2021.

### Study selection

Data were extracted from studies included in the review by two independent reviewers (SET and MM) using a standardized data extraction tool. First, the articles were selected based on their titles and abstracts. Second, full texts were evaluated to include or exclude the studies, and disagreements were resolved by consensus.

### Data collection and investigated outcomes

Data regarding authorship, year of publication, patient description, interventions (L-carnitine and control/), absolute numbers of each outcome, and follow-up period were extracted from the studies.

### Risk of bias and quality of evidence

The authors used the Risk of Bias (RoB 2)^19^^,^^20^ tool to assess the risk of bias for other fundamental elements, expressed as very serious, serious, or non-serious. The quality of the evidence was extrapolated from the risk of bias and was described by the Grading of Recommendations Assessment, Development and Evaluation (GRADE) terminology as very low, low, or high, and, for meta-analyses, it was described by the GRADEpro Guideline Development Tool (GDT; McMaster University, Hamilton, ON, Canada), as very low, low, moderate, or high.^21^

### Synthesis of results and analysis

Categorical outcomes were expressed by group (L-carnitine and placebo/), the number of events, and calculated relative risk (in %) for each group. The authors used the fixed-effect or random meta-analysis to evaluate the effect of L-carnitine versus placebo/ on the outcome when the data were available in at least two RCTs. The effects of meta-analyses were reported as the Risk Differences (RDs) and corresponding 95% CIs; a 95% CI including the number 0 in its range indicated that there was no difference in the outcome effect between the L-carnitine and control arms. The use of RD shows the absolute effect size in the meta-analysis when compared with relative risk or odds ratio, and this technique can be used when the binary outcome is zero in both study arms. The heterogeneity of effects among studies was quantified using the I2 statistic (I2 > 50% indicates high heterogeneity). In the case of high heterogeneity, the authors evaluated subgroups according to the L-carnitine dose in the mortality rate. For the meta-analysis, the authors used Review Manager software version 5.4.

## Results

**Fig. 1:**
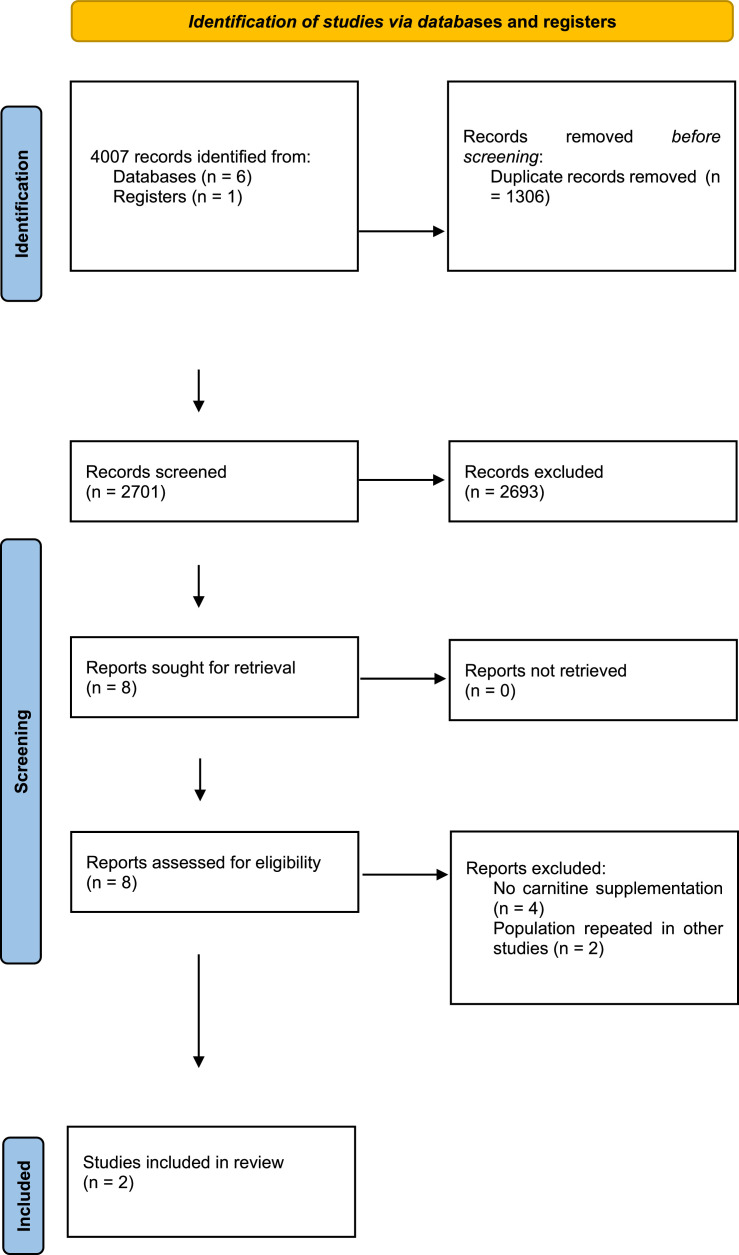
PRISMA flow diagram. Page MJ, McKenzie JE, Bossuyt PM, Boutron I, Hoffmann TC, et al. The PRISMA 2020 statement: An updated guideline for reporting systematic reviews BMJ 2021;372:n71. doi: 10.1136/bmj.n71.

**Fig. 2:**
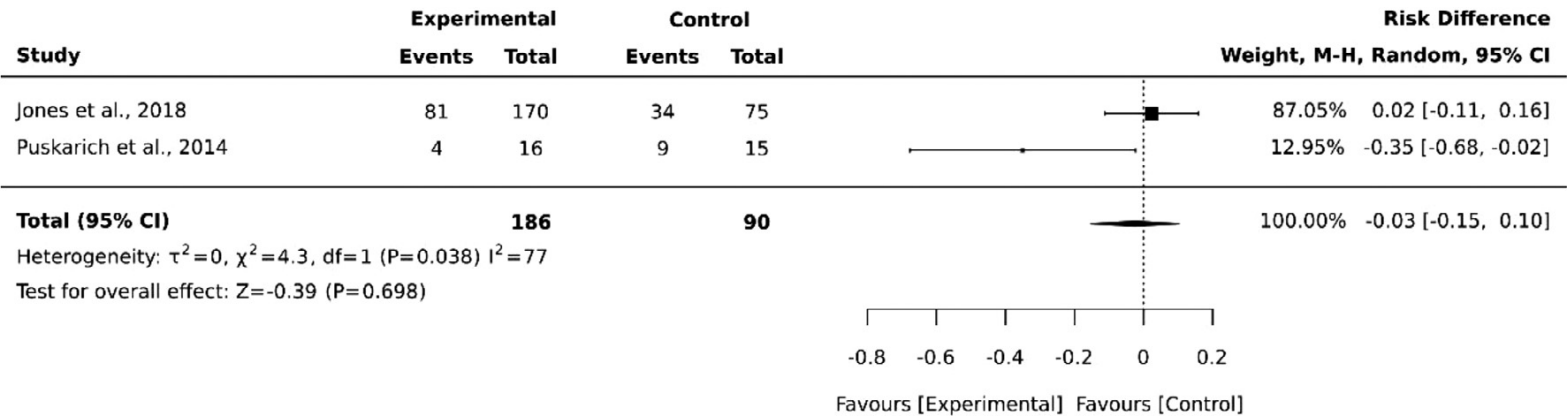
Forest-plot of L-carnitine versus placebo/usual care in 28-day mortality rate in hospitalized septic shock patients.

A total of 4,007 studies were retrieved from the selected databases (Fig. 1). After eliminating duplicates and including studies that satisfied the eligibility criteria, eight studies were selected for the assessment of their full texts. Of these, six studies were excluded. Therefore, two RCTs^21^, ^22^ were selected. The study characteristics are listed in Table 1. The authors considered a moderate risk of bias in the included studies (Table 2). For mortality outcomes, the authors considered 28 days, with 275 hospitalized patients with septic shock. No statistical difference was found in the L-carnitine vs. UC/placebo groups (DR = -0.03; 95% CI -0.15–0.10; I^2^ = 77%) (Fig. 2). The quality of evidence for the 28-day mortality rate was considered very low (Table 3). When the authors considered the subgroup analysis with intervention patients using 12g L-carnitine in both RCTs, we did not observe a statistical difference (DR = -0.06; 95% CI -0.24–0.12; I^2^ = 75%).

**Table 1:** Characteristics of included studies.

Study	Setting/ context	Participant characteristics	Groups	Outcomes measured	Description of main results
Jones AE, et al. 2018 USA	16 ICU	> 18y	Placebo	28-day mortality	Mortality in each group
		≤ 2 inflammatory criteria	Levocarnitine	SOFA change in 48h	
		≤ 24h septic shock	6g/12h	Hospital length	
		Use high dose vasopressors	12g/12h		
		SOFA > 6	18g/12h		
Puskarich MA, et al 2014 USA	1 ICU	> 18y	Placebo	28-day mortality	Mortality in each group
		Suspected infection	L-carnitine 12g/12h	Severe adverse event	
		SOFA > 5		SOFA change in 2 points in 24h and 48h	
		≥ 2 inflammatory criteria			
		≤ 24h septic shock			
		Use high dose vasopressors

**Table 2:** Risk of Bias 2 of included studies.

**Table 3:** L-carnitine compared to placebo/usual care on 28-days mortality rate in septic hospitalized patients.

Certainty assessment							N° of patients		Effect		Certainty study design	Importance risk of bias
N° of studies	Study design	Risk of bias	Inconsistency	Indirectness	Imprecision	Other considerations	Risk of bias	Inconsistency	Indirectness	N° of studies		
**Mortality (follow-up 28 days)**												
2^21^^,^^22^	Randomized clinical trials	Severe	Severe^a^	Not serious	Severe^b^	None	85/186 (45.7%)	43/89 (48.3%)	**RR 0.94** (0.71 to 1.23)	**29 fewer per 1.000** (from 140 fewer to 111 more)	⨁◯◯◯ Very Low^a^^,^^b^	Critical

CI, Confidence Interval; RR, Risk Ratio.

a High heterogeneity.

b Large variance of 95% CI.

## Discussion

This systematic review and meta-analysis demonstrated that the administration of L-carnitine to adult hospitalized septic shock patients did not significantly reduce the 28-day mortality.

L-carnitine supplementation is an interesting and innovative pharmacological approach to septic shock. Unlike other treatments, this strategy targets the metabolic derangements of sepsis. Although observational studies suggest that increased levels of L-carnitine and its esters are associated with decreased mortality in critically ill patients, this result was not observed in the two RCTs that were selected for this study.

Puskarich et al. performed a double-blind randomized control trial of L-carnitine infusion *vs* normal saline in 31 patients within 16h of recognition of septic shock. L-carnitine was administered as a 4g bolus injection over 2–3 minutes followed by an 8g infusion over the following 12h. The primary outcome was the proportion of patients demonstrating a decrease in the Sequential Organ Failure Assessment (SOFA) score of 2 or more points at 24h. Secondary outcomes were changes in SOFA score at 48h and 28-day mortality. The L-carnitine infusion was safe. There was no difference in the proportion of patients achieving a decrease in the SOFA score; however, 28-day mortality was slightly lower in the L-carnitine group.

The other RCT^22^ included in the analysis was an adaptive, double-blind, parallel-group trial that randomized 250 patients with septic shock. Patients were assigned to one of three active treatment arms: low (6 g), medium (12 g), and high (18 g) doses of levocarnitine administered intravenously or in the placebo group. Thirty-three percent of the dose was administered in bolus, followed by continuous infusion for the next 12h. The primary outcome was a change in the SOFA score from enrollment to 48h and 28-day mortality. Supplementation did not reveal beneficial results for these outcomes.

When summarizing these data, there was no difference in the 28-day mortality. This result was different from that of another meta-analysis that was published with L-carnitine supplementation in this scenario. A positive effect on mortality reduction in sepsis patients with the administration of L-carnitine was observed when the admission SOFA score was lower than 12. This effect was not observable in other subgroup analyses or in the overall effect. However, there is a methodological difference due to the inclusion of the same population twice in the meta-analysis, including intermediate outcomes. Therefore, the authors believe that this positive result could not make any clinical decision with a strong recommendation.

In the present study's own systematic review and meta-analysis, the authors aimed to achieve maximum sensitivity to identify all possibly relevant studies, as well as exclusively including high-quality RCTs with low risks of bias. The high heterogeneity identified in the meta-analysis can be related to the modification of the diagnosis of septic shock during RCT. This directly affects the selection of patients and their severity.

Although L-carnitine supplementation is an interesting approach that targets metabolic derangements of sepsis, a non-significant effect as a final result does not radically transform the current standard of care for septic shock patients. Even so, the authors deem that the lack of RCTs with this intervention in septic shock patients could have contributed to the negative results presented in this manuscript.

## Conclusions

In conclusion, there is low-quality evidence that the use of L-carnitine has no significant effect on reducing the 28-day mortality of septic shock patients.

## Abbreviations

GRADE, Grading of Recommendations Assessment, Development, and Evaluation; PICO, Patients of interest, intervention to be studied, comparison of intervention, and outcome of interest; RDs, Risk differences; RTC, Randomized clinical trials; SOFA, Sequential Organ Failure Assessment; UC, Usual Care.

## Ethics approval and consent to participate

Not applicable.

## Consent for publication

Not applicable.

## Availability of data and materials

Data sharing is not applicable to this article as no datasets were generated or analyzed during the current study.

## Authors’ contributions

MFM and SET contributed to the conception of the study, designed the meta-analysis, and extracted the data. SET performed the statistical analysis and data interpretation. GVG drafted the manuscript and developed the search strategy. MFM and SET revised the manuscript for important intellectual content, and all authors read and approved the final manuscript.

## Funding

FAPESP, process number 2020/01235-4.

## Conflicts of interest

The authors declare no conflicts of interest.
